# Molecular epidemiology of adenoviral keratoconjunctivitis in Saudi Arabia

**Published:** 2010-10-24

**Authors:** Khalid F. Tabbara, Nazri Omar, Ehab Hammouda, Masataka Akanuma, Takeshi Ohguchi, Toshihide Ariga, Yoshitsugu Tagawa, Nobuyoshi Kitaichi, Susumu Ishida, Koki Aoki, Hiroaki Ishiko, Shigeaki Ohno

**Affiliations:** 1 The Eye Center and The Eye Foundation for Research in Ophthalmology, Riyadh, Saudi Arabia; 2 Department of Ophthalmology, College of Medicine, King Saud University, Riyadh, Saudi Arabia; 3 The Wilmer Ophthalmological Institute and The Johns Hopkins University School of Medicine, Baltimore, MD; 4 Department of Ophthalmology, Faculty of Medicine and Health Sciences, Universiti Putra Malaysia, Serdang, Selangor, Malaysia; 5 Department of Ophthalmology and Visual Sciences, Hokkaido University Graduate School of Medicine, Sapporo, Japan; 6 Research and Development, Mitsubishi Kagaku Bio-Clinical Laboratories, Tokyo, Japan; 7 Department of Ocular Inflammation and Immunology, Hokkaido University Graduate School of Medicine, Sapporo, Japan

## Abstract

**Purpose:**

Adenoviral keratoconjunctivitis is a major cause of ocular morbidity and may lead to visual loss. Adenovirus types 8, 19, and 37 may cause epidemic keratoconjunctivitis. The main objective of this study was to determine the types of adenoviruses causing keratoconjunctivitis in Saudi Arabia.

**Methods:**

We conducted a non-interventional observational clinical study. Seventy three eyes from 65 patients who presented to The Eye Center in Riyadh, Saudi Arabia with clinical features of acute adenoviral keratoconjunctivitis were included. Each patient underwent complete clinical examination and features such as membranous reaction, conjunctival hemorrhage, subepithelial corneal infiltrates, and preauricular lymph node enlargement were recorded. Conjunctival swabs were obtained from patients with presumed acute viral conjunctivitis. Immunochromatography (IC) and restriction fragment length polymorphism polymerase chain reaction (PCR-RFLP) were performed on the conjunctival swabs obtained from each eye. Serotype identification was performed using direct sequencing technique.

**Results:**

Forty-nine (67.1%) were adenovirus type 8, 8 (11.0%) were adenovirus type 3, 6 (8.2%) type 37, 5 (6.8%) were adenovirus type 4, and 2 (2.3%) type 19. The remaining 5 were types 14, 19, and 22. The prevalence of membranous conjunctivitis was highest (83%) among eyes with adenovirus type 37 while subepithelial corneal opacities were most commonly seen among eyes with adenovirus type 8 (47%). Immunochromatography tests were positive for adenovirus in 48 (65.7%) out of 73 eyes.

**Conclusions:**

This study determined the types of adenoviruses causing keratoconjunctivitis at one center in Saudi Arabia. Direct sequencing techniques is an efficient, accurate, and rapid means of diagnosing adenoviral keratoconjunctivitis. The most common causes of adenoviral keratoconjunctivitis in Saudi Arabia were adenovirus types 8, 3, and 37. Membranous conjunctivitis and subepithelial opacities had the highest frequency of adenovirus types 37 and 8, respectively. Lymph nodes enlargement was least likely in adenovirus type 4.

## Introduction

Adenoviral keratoconjunctivitis is a common viral infection of the ocular surface and has a worldwide distribution. Certain adenovirus serotypes are non-pathogenic but a few infection in the eye can be in the form are associated with clinical diseases [1,2]. Adenoviral infection in the eye can be in the form of epidemic keratoconjunctivitis (EKC), pharyngoconjunctival fever, and non-specific conjunctivitis [3].The most common adenoviral serotypes that cause epidemic keratoconjunctivitis in order of frequency, are adenovirus types 8, 19, 37, and 5. The clinical manifestations of the disease may vary. For an instance, certain virus types cause corneal involvement and affect vision while others cause conjunctivitis without corneal involvement. In general, the diagnosis of adenoviral keratoconjunctivitis is made clinically but often there is conjunctival edema along with follicular reaction and hemorrhage [4]. Patients may also experience itching, pain and discharge. In severe cases, inflammatory membrane and conjunctival hemorrhage may develop. Corneal involvement gives rise to blurring of vision.

Saudi Arabia, as a host country for the Muslim pilgrims performing Hajj and Omra, becomes the destination of people from all over the world particularly during certain seasons of the year. This has contributed various health impacts to the local population. One of the effects was wide range of infectious organisms including the adenoviral keratoconjunctivitis. The serotypes of adenovirus are thought to be different as varied clinico-pathological manifestations were observed. To date, there has been no study to describe the clinical manifestations of adenoviral keratoconjunctivitis and their correlations with adenoviral serotypes in Saudi Arabia.

The main purpose of this study was to determine the adenovirus serotypes and clinical manifestations of adenoviral keratoconjunctivitis in Saudi Arabia.

## Methods

### Study design

A non-interventional observational clinical study.

### Patients

Seventy three eyes among 65 consecutive patients who were diagnosed with adenoviral keratoconjunctivitis based on its clinical features in the period of 2002 to 2007 were included in this study. This study was approved by the Institution Review Board (IRB) of The Eye Center, Riyadh, Saudi Arabia. Patients with clinical findings of sudden redness, discomfort, pain, tearing, conjunctival injection, follicular reaction, and tender pre-auricular lymph node enlargement and who had positive PCR for adenovirus were included. Epidemiological data obtained included registration number, age and gender. Each patient underwent complete biomicroscopic evaluation. Eye examinations were performed by one of us (KFT). Clinical data recorded comprised of presence or absence of conjunctival membranous reaction, conjunctival hemorrhage, corneal subepithelial infiltrate and preauricular lymphadenopathy. Patients were followed-up for a mean period of 14 months (range: 6 to 18 months).

Specimens from the inferior palpebral conjunctiva were obtained through conjunctival swabs. Specimens were stored at −70 °C until time of processing. Polymerase chain reaction (PCR) was done using restriction fragment length polymorphism (PCR-RFLP). Serotype identification was done using direct sequencing technique. Immunochromatography (IC) test was also performed on all conjunctival specimens.

### PCR technique and sequence analysis

Viral DNA was extracted from 100 µl of the conjunctival specimen with a Sumitest EX-R&D kit (Medical & Biologic Laboratories Co., Ltd., Nagano, Japan), according to the manufacturer's instructions. The extracted DNA was suspended in 100 µl of TE buffer (10 mM Tris, 1 mM EDTA, pH 8.0). PCR was performed in 50 µl of reaction mixture containing 5 µl of extracted viral DNA, 5 µl of 10× Taq buffer, 200 µM each of dATP, dGTP, and dTTP, 0.5 µM of each primer and 2.5 U of Taq DNA polymerase (TaKaRa EX-Taq, Takara Bio Inc., Shiga, Japan). After initial denaturation at 94 °C for 5 min, 40 cycles of denaturing at 94 °C for 30 s, annealing at 50 °C for 30 s, and extension at 72 °C for 1 min, a final extension at 72 °C for 7 min was performed using a Gene Amp PCR System 9600 (Applied Biosystems, Foster City, CA). The primers for amplication of the entire hexon were AdVID (nucleotides 17, 751–17, 772 of GenBank AF108105, 5′-TGT ATG TGC CTT ACG GCC AGA G-3′) and aDl3d2 (nucleotides 20,642–20,611) of GenBank AF108105, 5′-GCG CWC GAT GGR CGC RAG CT-3′). The primers for amplification of the entire fiber were AdD1 (nucleotides 5–25 of GenBank U69132, 5′-GAT GTC AAA TTC CTG GTT CCA C-3′) and aDd2 (nucleotides 1196–1198 of GenBank U69132, 5′-TAC CCG TGC TGG TGT AAA AAT C-3′). The positions of the primers for PCR were numbered according to the complete nucleotide sequence of the HAdV-2 strain (GenBank J01917).

The PCR products from the hexon gene and fiber gene were separated in 1% agarose gel and purified with a QIAquick Gel Extraction Kit (Qiagen, Valencia, CA). The nucleotide sequences of the entire hexon genes and the entire fiber genes were determined with a CEQ 2000XL DNA Analysis System with Dye Terminator Cycle Sequencing Kit (Beckman Coulter, Fullerton, CA). The primers used for cycle sequencing were the same as those used for the PCR that is described above.

### Immunochromatography test (IC)

The immunochromatography test (Adeno Test, SA Scientific, San Antonio, TX) was performed according to the manufacturer's instruction. Using a sterile swab, conjunctival specimen was taken from the lower palpebral conjunctiva without anesthesia. This was followed immediately by a short extraction step using a buffer solution. Using the specimen pipettes provided, four drops of the specimen solution were placed into the specimen well of the kit. Time was allowed for the specimen to filter through the kit to the specimen position and the control position. Appearance of a colored line at each position was looked for. The test was considered to be positive if lines were visible at the specimen and control positions. The test was considered negative if line was visible only at the control position. If no line was visible, the test was considered invalid and repeat test was performed for the same specimen. Readings were finalized not more than thirty minutes from application of specimen to the kit. A consent was obtained from each patient and Institution Review Board (IRB) approved this study.

## Results

### Age and gender distributions

The patients' age range was from 2 to 63 years with a mean age of 28.00±13.68 years. There were 33 (50.8%) males and 32 (49.2%) females in this study. Eight (5.2%) patients had bilateral involvement at presentation and both eyes were included separately in the study.

### Adenoviral serotypes

A total of 73 eyes of 65 patients showed positive PCR There were seven adenoviral serotypes identified causing adenoviral keratoconjunctivitis among the entire subjects in this study. Adenovirus (Ad) serotype 3 was found in 8 (11%) eyes, Ad4 in 5 (6.8%) eyes, Ad8 in 49 (67.1%), and Ad37 in 6 (8.2%). Other serotypes identified were Ad14 (1 eye, 1.4%), Ad19 (2 eyes, 2.7%), and Ad22 (2 eyes, 2.7%).

### Age and serotypes

Ad8 keratoconjunctivitis was distributed throughout all age groups. We noted that Ad3 and Ad4 did not occur in patients above 40 years while Ad37 did not occur in patient 20 years or less. There was no statistically significant association between the age and the different serotypes of adenoviral keratoconjunctivitis (λ2=10.68, p=0.56).

### Gender and serotypes

All adenoviral serotypes were distributed equally among both genders. There was no statistically significant association found between gender and serotypes of adenoviral keratoconjunctivitis (λ2=6.254, p=0.181).

### Adenotest and serotypes

**Figure 1:**
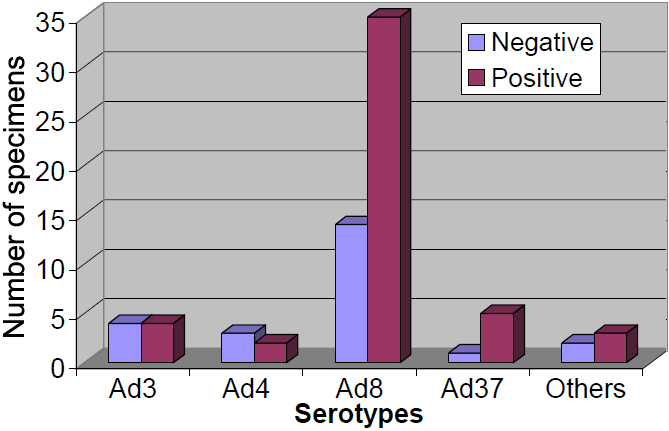
Distribution of immunochromatography result according to adenoviral serotypes among 73 ocular specimens.

Positive immunochromatography test rate was highest in adenoviral serotype 37 (83.3%) followed by Ad8 (71.43%), Ad3 (50%), and Ad4 (40%). There was no statistically significant difference between positive immunochromatography test result and different adenoviral serotypes in patients with keratoconjunctivitis (λ2=3.971, p=0.410). The distribution of adenoviral immunochromatography test result across different type of adenoviral serotypes is shown in Figure 1.

### Clinical features and serotypes

Corneal involvement in the form of subepithelial infiltrate was found in 30 (41.1%) eyes with keratoconjunctivitis. Highest prevalence was recorded in Ad8 with 48.98%. There was no statistically significant association between presence of cornea subepithelial opacity and adenoviral serotypes (λ2=5,032, p=0.284).

Membranous reaction was observed in 33 (45.2%) out of 73 eyes. Highest prevalence of membranous conjunctivitis among the adenovirus serotypes was recorded in Ad37 with 83.33%. There was no statistically significant association between membranous reaction and adenoviral serotypes in our study population (λ2=8.760, p=0.067).

Conjunctival hemorrhage was a feature in 42 (57.5%) eyes. Although prevalence was recorded highest in Ad8 (65%), there was no statistically significant association found between presence of conjunctival hemorrhage and serotypes of adenoviral keratoconjunctivitis (λ2=5.561, p=0.234).

Preauricular lymphadenitis occurred in 58 (79.5%) eyes. Prevalence was recorded the lowest in Ad4 (20%). There was a statistically significant association between presence of lymphadenitis and serotypes of adenoviral keratoconjunctivitis (λ2=13.593, p=0.009).

The prevalence of various clinical features in different adenoviral serotypes are summarized in Table 1.

**Table 1:** Number of clinical features according to different adenovirus serotypes in keratoconjunctivitis eyes.

** **	**Adenoviral (Ad) serotypes**				
**Clinical features**	**Ad 3 (%) [n=8]**	**Ad 4 (%) [n=5]**	**Ad 8 (%) [n=49]**	**Ad 37 (%) [n=6]**	**Other (%) [n=5]**
Corneal involvement	1 (12)	1 (20)	24 (49)	2 (33)	2 (40)
Membrane	1 (12)	1 (20)	23 (47)	5 (83)	3 (60)
Conjunctival hemorrhage	3 (38)	3 (60)	32 (65)	3 (50)	1 (20)
Lymph node swelling	6 (75)	1 (20)	43 (88)	4 (67)	4 (80)

## Discussion

Adenoviruses are large unenveloped double-stranded DNA viruses known to cause diseases of the respiratory tract, eyes, gastrointestinal tract and genitourinary tract in human. To date, there have been over 50 antigenically distinct adenovirus serotypes discovered based on neutralization techniques. They are subdivided into six subgenera A-F according to their ability to agglutinate red blood cells and their DNA homology [5-7].

Adenovirus detection can be performed using several methods. The gold standard is virus isolation followed by observation of cytopathic effect, in susceptible cell line. Although isolation of virus is definitive and allows further characterization, it is considered costly and time consuming. Culture was reported to take a range of 3 to 29 days with mean of 9.1 days [2,7,8]. Saitoh-Inagawa et al. [9] reported that it took three days to identify adenovirus serotypes by PCR-RFLP as compared to at least three weeks by culture isolation and neutralization test. Shell vial technique is a cell culture method that uses centrifugation and immunofluorescence to shorten culture positive time. In addition to being definitive, it provides positive results in shorter time than in the conventional cell culture [10].

Immunochromatography (IC) and enzyme immunoassay (EIA) for adenovirus have been described with variable sensitivity and specificity. Uchio et al. [11] reported sensitivity of 54% and specificity of 97.1% for IC. On the other hand, the sensitivity and specificity of EIA were reported to be 50.5% and 100%, respectively. While both tests were easier to be conducted, IC has the added advantage of providing faster result [8,11]. The PCR with the advantages of faster, more accurate and improved sensitivity, has been the method of choice lately. Sensitivity and specificity of the newer multiplex PCR has been shown to approach 100%. However, it has the disadvantages of high technical and expertise demand apart from high cost [2,3,8,11,12,13]. PCR-RFLP has the disadvantage of inability to identify adenovirus serotypes if a mutation in the restriction enzyme-cutting sites occur. PCR-sequencing may be useful to obtain more accurate serotyping [14,15].

Adenoviral conjunctivitis is largely caused by adenovirus serotypes 3, 4, 8, 19, 22 and 37 [9, 10,15,16]. EKC is commonly caused by subgroup D Adenovirus particularly serotype 8, 19, and 37, often without systemic syndrome association [2,3,8,16]. Adenovirus serotypes 3, 4, 7, and 11 cause mild conjunctivitis with systemic involvement. Adenovirus serotype 4 (Ad4) has broad symptomatology ranging from pharyngoconjunctival fever to adenoviral keratoconjunctivitis [17]. Ad4 has also been regarded as one of the major isolates in the adenoviral keratoconjunctivitis [18].

Jin et al. [19] conducted a study to investigate the epidemiology of adenoviral conjunctivitis in Hanoi, Vietnam and reported predominantly high prevalence of Ad8 (n=11, 78.6%) from fourteen PCR-positive conjunctival specimens. Other adenoviral serotypes found in their study were Ad3 (n=2, 14.3%) and Ad37 (n=1, 7.1%) [19]. Aoki et al. [20] reported prevalence of Ad8 in 124 (63.26%), Ad3 in 24 (12.24%), Ad4 in 33 (16.84%), and Ad19 in 15 (7.65%) of 196 PCR positive conjunctival specimens. Ishii et al. [21] compared the clinical and viral data of adenoviral keratoconjunctivitis in three east Asian cities, found Ad8 as the main serotypes in Sapporo (Japan), Koahsiung (Taiwan), and Busan (Korea). Other serotypes included Ad3, Ad4, Ad11, Ad19, and Ad37; each of which encountered for less 10% of cases [21].

Saitoh-Inagawa [22] in a study in Sapporo over 10 year period found Ad4 as the most common serotype and Ad3, Ad8, Ad11, Ad19, and Ad37 occurred less frequently. We experienced the similar pattern of adenovirus serotypes distribution among our patients. Ad8 was found to be the most frequent serotypes (49 eyes, 67.1%) in our study although the rate of Ad19 keratoconjunctivitis was relatively low (2 eyes, 2.3%).

IC for adenoviral in this study has been shown to be positive in 67% of PCR positive specimens. All specimens positive for IC were also positive for PCR which reflect high specificity. These finding are consistent with those reported by Jin et al. [19] and Levent et al. [23]. Adeno-check was developed for rapid diagnosis of human adenovirus by immunochromatography. However, it had low sensitivity although specificity was high. A later version, modified Adeno-check, was introduced to improve this drawback. Toshihide [24] compared the regular and modified versions regarding the sensitivity against adenoviral culture positive specimen from the conjunctiva. Regular version of Adeno-check showed 33 samples (48.5%) as positive compared to modified version which turned to be positive in 50 (73.5%) samples (p<0.01). They concluded that modified version was more sensitive than the regular version in the detection of human adenovirus [24].

Although adenovirus serotypes 3, 7, and 11 were associated with pharyngoconjunctival fever in children and serotypes 8, 19, and 37 were associated with epidemic keratoconjunctivitis affecting adults, we did not find that age was significantly associated with adenoviral serotypes among our keratoconjunctivitis patients. This may be explained partly by our small sample size and the possibility that patients with pharyngoconjunctival fever presented themselves to the physician for the systemic symptoms.Gender was not associated with adenoviral serotypes in adenoviral keratoconjunctivitis in our study population, consistent with the finding reported by Montessori et al. [25].

Conjunctival hemorrhage was reported to be present in 51.5% of PCR-proven conjunctivitis caused by Ad37 (68.1%) and Ad19 (19.2%) [12]. We found conjunctival hemorrhage in 57.5% eyes in Ad37 and Ad19. Lymphadenopathy was the single most important clinical sign in association with different adenovirus serotype while inflammatory membrane formation was less common. Other clinical features investigated in this study were not significantly associated with specific adenoviral serotypes.

Immunochromatography test using commercially available kit was not a satisfactory office detection for adenovirus keratoconjunctivitis. Ad8 was the major serotype causing adenoviral keratoconjunctivitis in Saudi Arabia. Corneal involvement and conjunctival hemorrhage were frequent in Ad8 and membranous reaction in Ad37. Larger multi-center study is necessary to asses the clinicopathology of adenoviral serotypes in keratoconjunctivitis. Subjects come to Saudi Arabia from various regions around the world and may bring with them viruses such as adenovirus. This may have contributed to the spread of epidemics of adenoviral keratoconjunctivitis.

## References

[1] Craighead JE. Adenoviruses In. Pathology and Pathogenesis of Human Viral Diseases. Academic Press: 2000. p. 189–201.

[2] Sambursky RP Fram N Cohen EJ The prevalence of adenoviral conjunctivitis at the Wills Eye Hospital Emergency Room. Optometry 2007 78 236 9 17478342 10.1016/j.optm.2006.11.012

[3] Percivalle E Sarasini A Torsellini M Bruschi L Antoniazzi E Grazia Revello M Gerna G A comparison of methods for detecting adenovirus type 8 keratoconjunctivitis during nosocomial outbreak in a Neonatal Intensive Care Unit. J Clin Virol 2003 28 257 64 14522064 10.1016/s1386-6532(03)00011-8

[4] Majeed A Naeem Z Khan DA Ayaz A Epidemic adenoviral conjunctivitis report of an outbreak in a military garrison and recommendations for its management and prevention. J Pak Med Assoc 2005 55 273 5 16108508

[5] Huang ML Nguy L Ferrenberg J Boeckh M Cent A Corey L Development of multiplexed real-time quantitative polymerase chain reaction assay for detecting human adenoviruses. Diagn Microbiol Infect Dis 2008 62 263 71 18707838 10.1016/j.diagmicrobio.2008.06.009 PMC2597193

[6] Cusack S Adenovirus complex structures. Curr Opin Struct Biol 2005 15 237 43 15837184 10.1016/j.sbi.2005.03.004

[7] Cheung D Bremner J Chan CTK Epidemic keratoconjunctivitis - do outbreaks have to be epidemics? Eye 2003 7 356 63 12724699 10.1038/sj.eye.6700330

[8] Weitgasser U Haller EM El-Shabrawi Y Evaluation of polymerase chain reaction for the detection of adenoviruses in conjunctival swabs specimens using degenerate primers in comparison with direct immunofluorescence. Ophthalmologica 2002 216 329 32 12424397 10.1159/000066175

[9] Saitoh-Inagawa W Oshima A Aoki K Itoh N Isobe K Uchio E Ohno S Nakajima H Hata K Ishiko H Rapid diagnosis of adenoviral conjunctivitis by PCR and restriction fragment length polymorphism analysis. J Clin Microbiol 1996 34 2113 6 8862567 10.1128/jcm.34.9.2113-2116.1996 PMC229199

[10] Kowalski RP Keranchak RM Romanowski EG Gordon YJ Evaluation of the shell vial technique for detection of ocular adenovirus. Community Ophthalmologists of Pittsburgh, Pennsylvania. Ophthalmology 1999 106 1324 7 10406615 10.1016/s0161-6420(99)00718-6

[11] Uchio E Aoki K Saitoh W Itoh N Ohno S Rapid diagnosis of adenoviral conjunctivitis swabs by 10-minute Immunochromatography. Ophthalmology 1997 104 1294 9 9261316 10.1016/s0161-6420(97)30145-6

[12] Van Gelder RN Applications of the polymerase chain reaction to diagnosis of ophthalmic diseases. Surv Ophthalmol 2001 46 248 58 11738432 10.1016/s0039-6257(01)00274-0

[13] Chang CH Sheu MM Lin KH Chen CW Hemorrhagic viral keratoconjunctivitis in Taiwan caused by Adenovirus types 19 and37: applicability of polymerase chain reaction-restriction fragment length polymorphism in detecting adenovirus genotypes. Cornea 2001 20 295 300 11322419 10.1097/00003226-200104000-00011

[14] Takeuchi S Itoh N Uchio E Aoki K Ohno S Serotyping of adenoviruses on conjunctival scrapings by PCR and sequence analysis. J Clin Microbiol 1999 37 1839 45 10325334 10.1128/jcm.37.6.1839-1845.1999 PMC84965

[15] Matsui K Saha S Saitoh M Mizuki N Itoh N Okada E Yoshida A Xin KQ Nishio O Okuda K Isolation of adenovirus from conjunctival scarapings over a two-year period (between 2001 and 2003) in Yokohama, Japan. J Med Virol 2007 79 200 5 17177307 10.1002/jmv.20779

[16] Hamada N Gotoh K Hara K Iwahashi J Imamura Y Nakamura S Taguchi C Sugita M Yamakawa R Etoh Y Sera N Ishibashi T Chijiwa K Watanabe H Nosocomial outbreak of epidemic keratoconjunctivitis accompanying environmental contamination with adenoviruses. J Hosp Infect 2008 68 262 8 18289721 10.1016/j.jhin.2007.12.012

[17] Aoki K Tagawa Y A twenty-one year surveillance of adenoviral conjunctivitis in Sapporo, Japan. Int Ophthalmol Clin 2002 42 49 54 12189615 10.1097/00004397-200201000-00008

[18] Ariga T Shimada Y Ohgami K Tagawa Y Ishiko H Aoki K Ohno S New genome type of adenovirus serotype 4 caused nosocomial infections associated with epidemic conjunctivitis in Japan. J Clin Microbiol 2004 42 3644 8 15297510 10.1128/JCM.42.8.3644-3648.2004 PMC497636

[19] Jin XH Okamoto Y Morishita J Tsuboi K Tonai T Ueda N Molecular epidemiology of adenoviral conjunctivitis in Hanoi, Vietnam. Am J Ophthalmol 2006 142 1064 6 17157595 10.1016/j.ajo.2006.07.041

[20] Aoki K Kato M Ohtsuka H Ishii K Nakazono N Sawada H Clinical and etiological study of adenoviral conjunctivitis, with special reference to adenovirus types 4 and 19 infections. Br J Ophthalmol 1982 66 776 80 6293531 10.1136/bjo.66.12.776 PMC1039927

[21] Ishii K Nakazono N Fujinaga K Fujii S Kato M Ohtsuka H Aoki K Chen CW Lin CC Sheu MM Lin KH Oum BS Lee SH Chun CH Yoshii T Yamazaki S Comparative studies on aetiology and epidemiology of viral conjunctivitis in three countries of East Asia and Japan, Taiwan and South Korea. Int J Epidemiol 1987 16 98 103 3032816 10.1093/ije/16.1.98

[22] Saitoh-Inagawa W Aoki K Uchio E Itoh N Ohno S Ten years' surveillance of viral conjunctivitis in Sapporo, Japan. Graefes Arch Clin Exp Ophthalmol 1999 237 35 8 9951639 10.1007/s004170050191

[23] Levent F Greer JM Snider M Demmler-Harrison GJ Performance of a new immunochromatographic assay for detection of adenoviruses in children. J Clin Virol 2009 44 173 5 19101199 10.1016/j.jcv.2008.11.002

[24] Ariga T Miura R Tagawa Y Yohashi T Nakagawa H Okamoto S Hinokuma R Asato Y Kaneko H Ishiko H Aoki K Ohno S Clinical evaluation of modified adeno-check. Rinsho Ganka 2005 59 1183 8

[25] Montessori V Scharf S Holland S Werker DH Roberts FJ Bryce E Epidemic keratoconjunctivitis outbreak at a tertiary referral eye care clinic. Am J Infect Control 1998 26 399 405 9721392 10.1016/s0196-6553(98)70035-5

